# Coordinated responses of natural anticoagulants to allogeneic stem cell transplantation and acute GVHD – A longitudinal study

**DOI:** 10.1371/journal.pone.0190007

**Published:** 2017-12-22

**Authors:** Beata Przybyla, Anne Pinomäki, Jari Petäjä, Lotta Joutsi-Korhonen, Karin Strandberg, Andreas Hillarp, Ann-Kristin Öhlin, Tapani Ruutu, Liisa Volin, Riitta Lassila

**Affiliations:** 1 Coagulation Disorders Unit, Helsinki University Hospital and University of Helsinki, Helsinki, Finland; 2 Stem Cell Transplantation Unit, Helsinki University Hospital Comprehensive Cancer Center, Helsinki, Finland; 3 Department of Paediatrics, Helsinki University Hospital, Helsinki, Finland; 4 Hematology and Clinical Chemistry and HUSLAB Laboratory Services, Helsinki University Hospital, Helsinki, Finland; 5 Department of Clinical Chemistry, Lund University, Lund University Hospital, Lund, Sweden; 6 University and Regional Laboratories, Skane County Council, Coagulation Laboratory, Clinical Chemistry, Malmö, Sweden; University of Navarra School of Medicine and Center for Applied Medical Research (CIMA), SPAIN

## Abstract

**Background:**

Allogeneic stem cell transplantation (SCT) enhances coagulation via endothelial perturbation and inflammation. Role of natural anticoagulants in interactions between coagulation and inflammation as well as in acute graft-versus-host disease (GVHD) are not well known. The purpose of this study was to define changes in natural anticoagulants over time in association with GVHD.

**Patients and methods:**

This prospective study included 30 patients who received grafts from siblings (n = 19) or unrelated donors (n = 11). Eight patients developed GVHD. Standard clinical assays were applied to measure natural anticoagulants, represented by protein C (PC), antithrombin (AT), protein S (PS), complex of activated PC with its inhibitor (APC-PCI) and by markers of endothelial activation: Factor VIII coagulant activity (FVIII:C) and soluble thrombomodulin (s-TM) at 6–8 time points over three months.

**Results:**

Overall, PC, AT and FVIII:C increased in parallel after engraftment. Significant correlations between PC and FVIII:C (r = 0.64–0.82, p<0.001) and between PC and AT (r = 0.62–0.81, p<0.05) were observed at each time point. Patients with GVHD had 21% lower PC during conditioning therapy and 55% lower APC-PCI early after transplantation, as well as 37% higher values of s-TM after engraftment. The GVHD group had also increases of PC (24%), FVIII: C (28%) and AT (16%) three months after transplantation.

**Conclusion:**

The coordinated activation of natural anticoagulants in our longitudinal study indicates the sustained ability of adaptation to endothelial and inflammatory activation during allogenic SCT treatment. The suboptimal control of coagulation by natural anticoagulants at early stage of SCT may contribute to onset of GVHD.

## Introduction

Allogeneic SCT is the most efficient, and often the only curative treatment modality in various, usually malignant hematological disorders. The SCT includes conditioning, graft infusion, engraftment and recovery. Conditioning therapy is used to eradicate hematological cancers, but is also mandatory to avoid rejection of the graft. This therapy causes vascular injury [1] leading to activation of coagulation. Bursts of coagulation and fibrinolysis during conditioning and at later stages of allogeneic SCT have been previously described [2,3].

Coagulation is controlled by natural anticoagulants such as PC and AT. Thrombin, the key activator of coagulation pathway, activates PC by binding to endothelial TM. APC aided by cofactor PS cleaves coagulation cofactors V/Va and VIII/VIIIa slowing down the production of thrombin. APC activity carries also anti-inflammatory, cytoprotective and anti-apoptotic properties [4–6]. AT inhibits thrombin and other coagulation factors by binding active site, and this process is enhanced by endothelial heparan sulphate.

Under physiological conditions, endothelium keeps its anticoagulant state. During damage, activated endothelium releases s-TM with the local function to control activation of PC. Though deficiencies and changes of natural anticoagulants have been reported in patients following autologous and allogeneic SCT [7–12], the contribution of this regulatory system to clinical outcome of SCT is still poorly understood.

Graft-versus-host disease (GVHD), a major complication of SCT, is caused by immune reaction of donor T cells towards patient’s organs. Acute GVHD usually occurs before, and chronic GVHD after around 3 months following transplantation. The early endothelial damage occurring during conditioning regimen contributes to pathogenesis of GVHD [13,14]. Endothelial cells, activated during conditioning contribute to propagation of coagulation and inflammation. Pro-inflammatory cytokines [15–17] are important for perpetuated inflammation and endothelial damage [1,18,19]. Cytokine-stimulated endothelial cells express tissue factor, which initiates and amplifies blood coagulation interacting with inflammation. Endothelial switch from an anticoagulant to procoagulant state associates with loss of glycocalicin and initiation of thrombin and fibrin generation. Fibrin in turns recruits both platelets and more inflammatory cells, which perpetuating the damage. Therefore, monitoring the evolution of natural anticoagulant responses together with endothelial damage over time after SCT may help to define more factors that contribute to aGVHD.

Our previous allogeneic SCT study was focused on coagulation and fibrinolysis. We found that development of acute GVHD associated with increase of thrombin generation and decrease of fibrinolysis at early stages of the treatment [3]. In the present study, we report longitudinal analyses of natural anticoagulants: AT, PC and its cofactor PS, APC-PCI complex, as well as markers of endothelial activation: FVIII: C and s-TM. We focus on the associations between temporal changes of natural anticoagulants and development of GVHD, noting the limitations of the study size and confounders, such as donor type and infections.

## Patients and methods

### Patients

Thirty patients undergoing myeloablative allogeneic SCT for a hematological malignancy took part in this prospective study [3]. The study was approved by the Ethics Committee of the Helsinki University Hospital, and a written informed consent was obtained from each patient. Laboratory monitoring lasted for 3 months and clinical monitoring for the median of 40 months. 19 patients received transplants from HLA-identical siblings and 11 patients from matched unrelated donors. Stem cells in 19 cases were harvested from bone marrow and in 11 cases from peripheral blood. The myeloablative conditioning regimen consisted of Cyclophosphamide 60 mg/kg once daily i.v. on days -6 and -5 and of total body irradiation (12.0 Gy) in six fractions (lungs 10 Gy) on days -4 to 0 in all patients. The graft was infused on the day 0. Cyclosporine and a short course of Methotrexate was given as GVHD prophylaxis. If the donor was unrelated, 2 mg/kg/day of antithymocyte globulin (ATG; Thymoglobuline^®^; Sangstat, Lyon, France) was administrated on days -3 to -1. Patients receiving grafts from siblings received methylprednisone (MP) as GVHD prophylaxis from the day +eight, as described [20]. Acute GVHD was assessed according to published criteria [21], and treated with MP 2–10 mg/kg/day divided into 4 i.v. doses upon appearance [3].

### Blood collection and plasma samples analyses

Blood samples were collected before (d -10), and during (d-2) conditioning therapy, early after transplantation (d+10), after engraftment (d +24), at d+38, d +52 and at the end of the study (d+90). Blood was collected in the morning, always before possible red cell and platelet transfusions. Samples for coagulation assays were obtained in 109 mM (3.2%) trisodium citrate from antecubital veins using brief stasis only. After immediate transfer to laboratory, plasma was isolated by centrifugation for 10 min at 2000 g at 22°C. Plasma aliquots were stored at -70°C if not tested immediately. Blood for APC-PCI assay was collected in special vacuum tubes (Biopool Stabilyte^™^, Trinity Biotech, Bray, Ireland) to inhibit the complex formation *in vitro* and platelet activation [22]. Blood collected in these tubes were centrifuged for 30 min at 1800 g at 4°C.

FVIII: C, PC and AT activities and free PS antigen were measured in the Helsinki University Hospital Laboratory using routine procedures: coagulometry (Siemens Healthcare AB)—FVIII:C; chromogenic assays—AT [(Berichrom^®^Anthithrombin III(A)] and PC (Berichrom^®^Protein C), both from Siemens, Marburg, Germany; and automated latex ligand immunoassay for PS (Instrumentation Laboratory, Lexington, MA, USA). The reference values were as follow: FVIII: C 52–148%, PC 74–141%, AT 84–108%, PS 66–150% (men) and 50–137% (women).

Antigen and activity of soluble TM (s-TM-Ag and s-TM-Act) were measured in Stabilyte plasma using an in-house method [23], with the detection limit 0.03 SEq mL^-1^ (+3 SD for the blank sample). In healthy individuals s-TM-Ag ranged 3.5–8.3 SEq mL^-1^ (men; n = 50) and 3.2–7.2 SEq mL^-1^ (women; n = 50) and s-TM-Act 2.1–5.7 SEq mL^-1^ (men) and 3.2–7.2 SEq mL^-1^ (women). The intra- and inter-assay coefficients of variation were <8% and <15%, respectively [22].

APC-PCI complex was measured using the immunochemical sandwich method [22]. The levels of APC-PCI in healthy individuals ranges between 0.07 and 0.26 μg L^-1^ with median equal 0.13 μg L^-1^ (n = 80). The within-run coefficient of variation (CV %) was 4.8% at 0.15 μg L^-1^ and 3.2% at 0.40 μg L^-1^, while the between-run CV % was 7.1% at 0.15 and 5.8% at 0.40 μg L^-1^ (n = 38).

### Statistical methods

Distribution of each variable was tested and an appropriate statistical approach was chosen. Changes in variables were evaluated with Friedman’s and Dunn’s multiple comparison tests. Analysis of variance (ANOVA) with repeated measures, the Mann Whitney and t-tests were used for group comparisons. Correlations were evaluated using Spearman’s rank correlation coefficients. Two-tailed *P*-values < 0.05 were considered statistically significant. Statistical calculations were performed using the SPSS for Windows version 15.0 (SPSS, Chicago, IL, USA).

## Results

### Temporal changes of endothelial activation markers and natural anticoagulants before and after allogeneic stem cells transplantation

**Fig 1:**
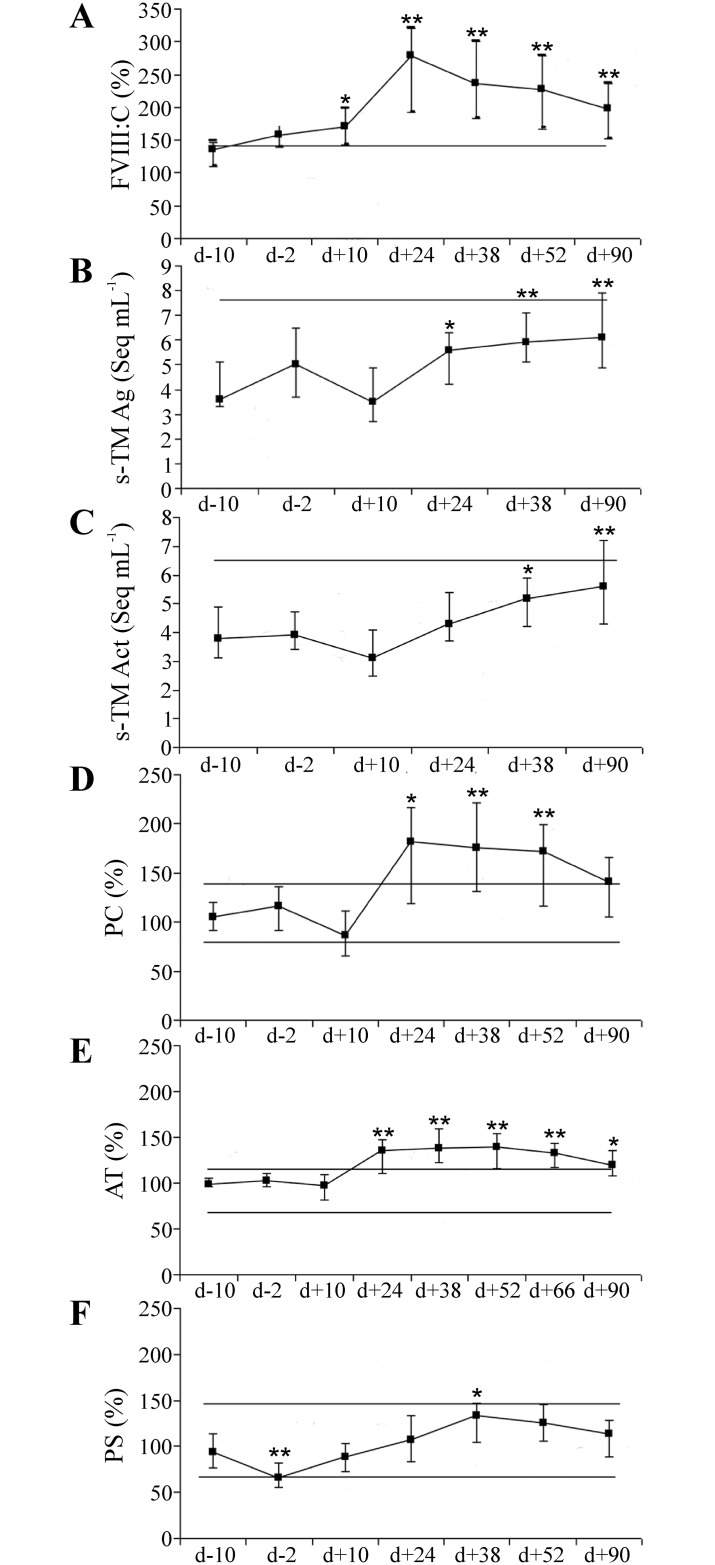
Longitudinal changes of endothelial activation markers and natural anticoagulants in allogenic SCT/graft infusion. Endothelial markers: (A) Factor VIII with coagulant activity (FVIII:C); (B) soluble thrombomodulin antigen (s-TM Ag); (C) soluble thrombomodulin activity (s-TM Act) are shown at 6–7 time points during allogenic SCT treatment. Activities of natural anticoagulants: (D) protein C; (E) antithrombin; and (F) protein S were measured at 7–8 time points. Data points represent median values and error bars—25^th^ and 75^th^ percentiles; comparisons were with baseline values. The horizontal line depicts lower and upper normal reference limits; d = transplantation /graft infusion day; * = p < 0.05; ** = p < 0.001.

As a marker of acute phase and endothelial activation the von Willebrand factor-bound FVIII (FVIII: C) was increasing after transplantation (d +10) until engraftment (d+24) and stayed elevated until the end of study (Fig 1A).

The s-TM antigen (s-TM Ag) which also manifests endothelial activation heightened in association with the engraftment. The activity of s-TM (s-TM Act) also increased after engraftment (d+24) and both remained up regulated but within range of normal references. After engraftment, s-TM antigen and activity were positively correlated (p < 0.05). The correlation coefficients between s-TM Ag and s-TM Act varied from 0.43 to 0.58. The natural anticoagulants PC and AT responded to changes of FVIII: C; the median values of both PC and AT followed the pattern of FVIII: C course (Fig 1A, 1D and 1E). After engraftment, both PC and AT increased approximately 50% and stayed elevated until the end of the study. The five-fold inter-individual variability of PC and FVIII: C was observed (Fig 1A and 1D). The PS, a cofactor of PC, decreased to low normal values during conditioning, but kept increasing up to the time point d +38 (Fig 1F). PC, AT and FVIII: C showed significant correlations throughout the study (Table 1).

**Table 1:** Correlations between PC, FVIII and AT at different stages afte allogenic graft infusion.

		Time points (d = transplantation day)						
		d –10	d—2	d + 10	d + 24	d + 38	d + 52	d + 90
PC vs FVIII:C	R	0.58	0.38		0.64	0.64	0.82	0.76
	p	0.001	0.037		< 0.001	< 0.001	< 0.001	< 0.001
PC vs AT	R	0.49	0.62	0.62	0.75	0.81	0.64	0.69
	p	0.007	0.01	0.01	0.01	0.01	0.01	0.01

R–Spearman’s rank correlation coefficient, p < 0.05

### APC-PCI complex changes over time

**Fig 2:**
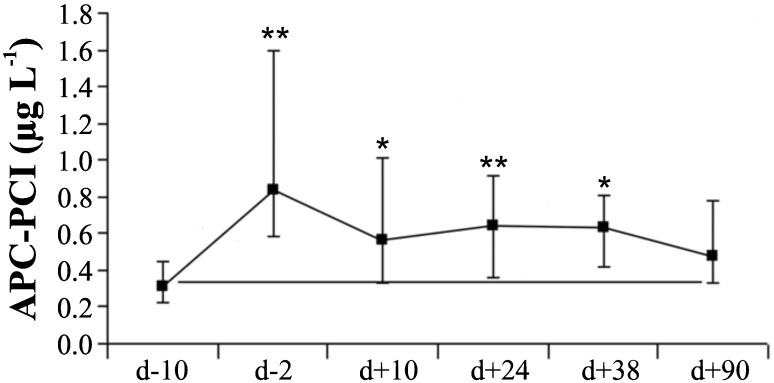
Variability of APC-PCI between different time points during allogenic SCT. Activated protein C complexed with its inhibitor (APC-PCI) was measured at 6 time points. Data are presented as median values and error bars as the 25^th^ and 75^th^ percentiles; comparisons were with baseline values; d = transplantation/graft infusion day; * = p < 0.05; ** = p < 0.001.

APC-PCI is a sensitive marker of natural anticoagulant system responding to thrombin generation. The concentration of circulating APC-PCI increased during the conditioning therapy and remained elevated (Fig 2).

In a previous study [3] of the same group of patients, we measured release of F1+2, a product of prothrombin cleavage indicating thrombin generation. We combined the APC-PCI and the F1+2 values to define a ratio F1+2/APC-PCI, which had the highest values (>3) at the day d+10 (data not shown).

### Onset of GVHD and temporal changes of natural anticoagulants in allogeneic SCT

**Fig 3:**
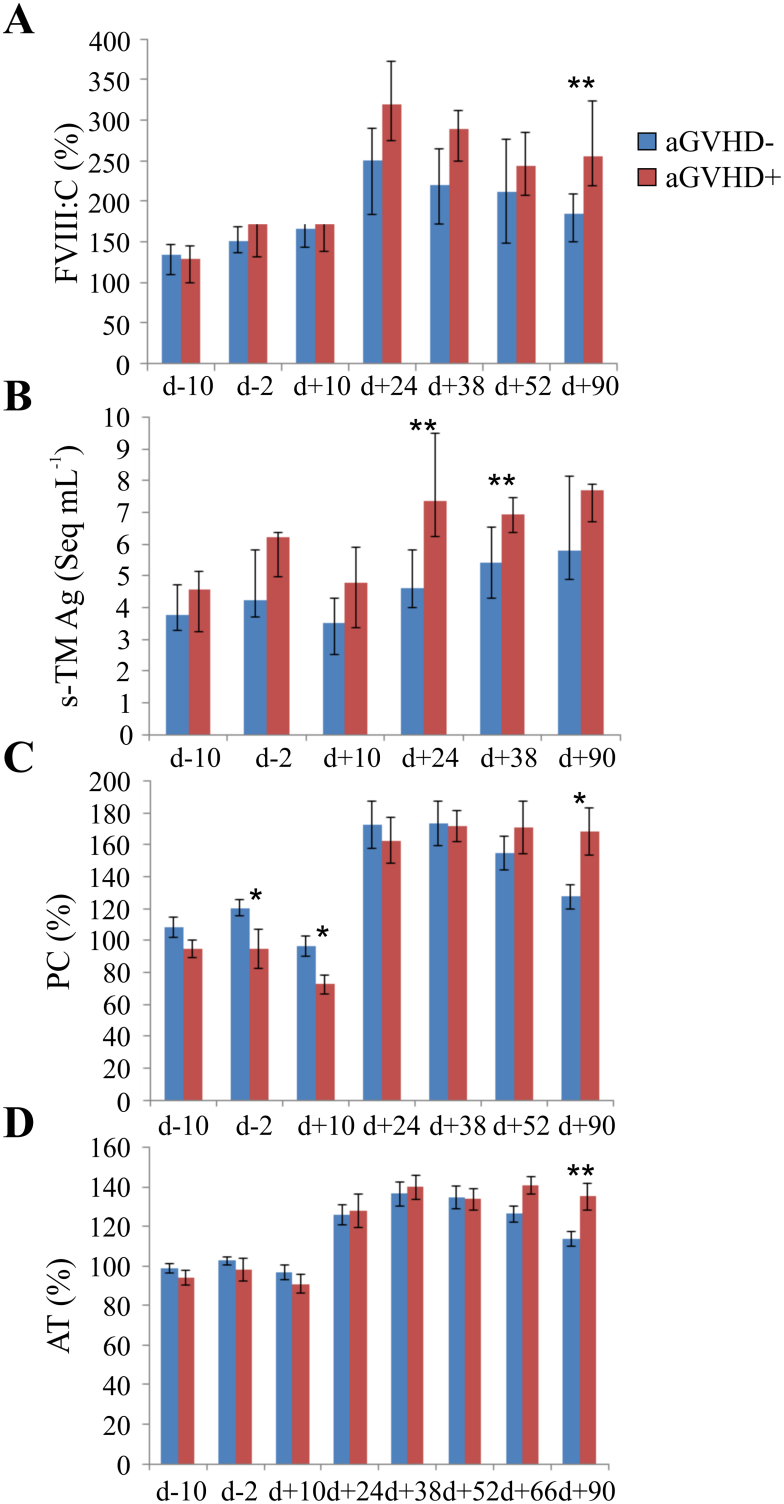
Natural anticoagulants and endothelial activation markers associated with aGVHD. Differences between the group of acute GVHD (aGVHD+, N = 8) and without GVHD (aGVHD-, N = 22), are depicted for (A) FVIII, (B) s-TM endothelial antigen, (C) protein C (PC) activity and (D) antithrombin. Data on panels (A) and (B) are presented as median values with error bars as the 25^th^ and 75^th^ percentiles; and on panels (C) and (D) as mean values and error bars as the standard errors; d = transplantation day; * = p < 0.05; ** = p < 0.01.

The patients were divided into two groups: without acute GVHD (n = 22) and with GVHD (n = 8). Four GVHD patients were recipients of grafts from unrelated donors (4/11 = 36%). The aGVHD in this group occurred between 11 and 68 days after transplantation (median 13 days). The four GVHD patients who obtained grafts from siblings showed the first GVHD symptoms later, between 44–75 days (median 44 days). The seven endothelial and coagulation variables were tested for associations with GVHD at all time points. Fig 3 presents comparisons between the groups with and without GVHD. Four variables had at least one time point showing significant differences between the groups. These were: FVIII:C, s-TM Ag, PC and AT. In the GVHD group, PC had significantly lower values at early time points d-2 and d+10, s-TM Ag was higher at d+24 and d+38, FVIII:C and PC at d+90, and AT at d+90.

Differences between a group with acute GVHD (aGVHD+, N = 8) and without acute GVHD (aGVHD-, N = 22), are depicted for (A) Factor VIII (B) s-TM endothelial antigen (C) protein C activity and (D) anthithrombin. Data on panels A and B d are presented as median values and error bars as the 25^th^ and 75^th^ percentiles; and on panels C and D as mean values with error bars representing standard errors. d = transplantation day; * = p < 0.05; ** = p < 0.01The significant increase of endothelial activation markers in the GVHD group was observed for s-TM Ag at 24 and 38 days and for FVIII:C at 90 days after transplantation (Fig 3A and 3B). The decrease of natural anticoagulant PC occurred at early time points, d-2 and d+10, but clearly elevated at the d+90 day. (Fig 3C). Again, a significant increase in s-TM Ag level occurred at d+90 in the GVHD group (Fig 3D).

## Discussion

The aim of this study was to define a role of natural anticoagulants in the adaptation to immune milieu during allogenic SCT. We have previously reported the activation of the coagulation system, manifested by thrombin generation and release of prothrombin fragment 1.2 (known as F1+2), during the conditioning therapy and later stages of allogeneic SCT [3]. In the present study, we have evaluated natural anticoagulants in the same blood samples. We found that the activated PC and complexed with PCI responded immediately to the thrombin challenge during conditioning therapy. Endothelial markers paralleled PC later following engraftment. We also found that low PC activity during the conditioning therapy predicted later development of acute GVHD.

The AT and PC activities were changing in parallel. This is somewhat surprising because PC and TM engage different inhibitory mechanisms. It is likely that AT and PC are carried by the same endothelial vesicles. It was shown previously that the TM-PC and the AT-heparan sulphate complexes are shed by endothelium into circulation [24]. Circulatory microvesicles play important role in interactions between inflammatory, immune and coagulation systems [25], and in regulation of sepsis (24).

Our longitudinal monitoring of protein C system in SCT patients allowed discovering a unique long-lasting balance between PC, APC and thrombin. Normally, acute thrombin generation causes rapid, a minute- scale rise of APC level [26]. The normal molar ratio of PC to APC in human plasma is approximately 2000:1 [27], securing a large reserve for rapid APC formation upon TM-dependent activation. Under prolonged thrombin formation and/or inflammatory challenges maintenance of PC-APC function may be compromised. For example, patients with sepsis develop PC deficiency caused by exhaustion and/or disturbed activation of PC precursor- zymogen in circulation [28]. Low or dysfunctional PC coincides with shedding of endothelial TM and poor prognosis in critically ill patients [29].

Alteration in integrity of vasculature contributes to GVHD [14]. The endothelial damage initiated by condition therapy perpetuates and manifests endothelial parameters alterations in later time points.

The analysis of protein C system in groups with- and without GVHD indicates that defective APC generation in response to thrombin may predispose to GVHD suggesting causal relationship between low PC during the conditioning therapy and later occurrence of GVHD. This low PC during preconditioning in the GVHD may result from a higher level of endothelial injury. We have also suggested a ratio F1+2/APC-PCI as a new parameter to monitor coagulation-anticoagulation “dysregulated” responses over time during SCT.

Endothelial damage, mediated by proinflammatory cytokines, underlies the pathogenesis of many complications after myeloablative allogeneic SCT [30]. Shedding of TM by damaged endothelium is manifested by an increased level of circulating s-TM after engraftment. Indeed, an earlier study found an association between GVHD and s-TM Ag [31]. Monitoring of s-TM in our patients showed that s-TM became functional at later time points during SCT recovery. Since the activation of PC by phospholipid-bound TM is much faster than by free TM [32], the contribution of s-TM to activation of PC in SCT throughout our time points, may depend on associating with microparticles in circulation. The increase of s-TM after engraftment was paralleled by increase of FVIII: C, reflecting endothelial activation. Our data agree with previously observed increase of von Willebrand factor (a carrier of FVIII) after transplantation [33]. Interestingly, recombinant s-TM appeared successful in the treatment of capillary leakage syndrome, an early complication after allogeneic SCT [34].

Our TM findings are in line with a published data related to TM and GVHD. The higher level of TM was observed in GVHD patients after umbilical cord blood transplantation [35]. A study of temporal changes of TM in myeloablative recipients reported by Cutler et al. [36] concluded that TM level higher than 100 ng/mL on the d+7 day predicted the occurrence of veno-oclusive disease (VOD). In a study of bone marrow transplantation [37] an increase of TM plasma levels at days 15 and 22 after conditioning therapy associated with clinical complications such as septicaemia, GVHD and VOD.

The present study focused on temporal changes of the seven variables of endothelial activity, blood coagulation and its regulation, and their differences between the groups either developing GVHD or not. We recognize that possible confounders, such as preventive drugs, draft source and donor type can contribute to our observations in our relatively small study.

In summary, our results revealed new interactive regulatory mechanisms activated in response to allogenic SCT and development of GVHD. These interactions occurred despite a limited number of patients with multiple confounding factors, such as different drugs, grafts, donor types and infections. The time course of TM and PC activities suggests that PC-APC system undergoes adaptation to vascular alterations and inflammation during conditioning, engraftment and recovery. Therein, we propose that degree of PC activity during preconditioning might be an indicator of future GVHD. Overall, monitoring of the coordination between variables of endothelial activation, coagulation and natural anticoagulants may lead to discoveries, which will alleviate complications of SCT.

## Supporting information

S1 Table Raw data. Raw data related to measurements of natural anticoagulants in plasma of 30 SCT patients. The columns contain the following data: presence of acute GVHD (1 = aGVHD+, 0 = aGVHD-), type of donor (1 = unrelated donor, 0 = siblings), type of graft (1 = peripheral blood, 0 = bone marrow), protein C (PC) (7 time points), FVIII:C (7 time points),antithrombin (AT) (8 time points), soluble thrombomodulin (s-TM) activity (Act) (6 time points), s-TM antigen (Ag) (6 time points), protein S (PS) (7 time points), activated PC (APC) and protein C inhibitor complex (APC-PCI) (7 time points).
(ZIP)
